# Chlorpromazine inhibits the plasmid-mediated *oqxAB* multidrug efflux pump in *Escherichia coli* isolates of Egyptian patients with utis

**DOI:** 10.1186/s12866-025-03850-7

**Published:** 2025-03-26

**Authors:** Kholoud Baraka, Rania Abozahra, Fatma Okda, Sarah M. Abdelhamid

**Affiliations:** Microbiology and Immunology Department, Faculty of Pharmacy, Damanhour University, El Gomhoreya Street, El Behira, Egypt

**Keywords:** *E. coli*, *OqxA*, *OqxB*, Quinolone, Conjugation

## Abstract

Over the past ten years, the prevalence of the OqxAB efflux pump, a plasmid-mediated quinolone resistance determinant, has increased among *Escherichia coli* (*E. coli*) isolates. The aim of this study was to genotypically and phenotypically investigate quinolone resistance of isolates and transferability of *oqxAB* genes by conjugation. One hundred *E. coli* isolates were collected from UTIs samples and identified using biochemical techniques and confirmed by VITEK-2 System. Antibiotic resistance of isolates was determined by disc diffusion method. MIC of levofloxacin was determined using the broth microdilution method. Efflux pump-mediated resistance was assessed using the chlorpromazine-based efflux-pump inhibitor microplate assay. PMQR genes (*oqxA*, *oqxB*) were detected by conventional PCR. A conjugation experiment was run to investigate the transferability of the quinolone resistance in having plasmids carrying *oqxAB*. DNA sequencing was performed for the identification of genes in both donors and tranconjugants. 80% of *E. coli* isolates were resistant for levofloxacin. Chlorpromazine significantly decreased the levofloxacin MIC values. *oqxA* and *oqxB* genes were detected in 44% and 39% of levofloxacin resistant isolates, respectively. The conjugation experiment revealed the transfer of resistance. MICs of levofloxacin in transconjugants carrying *oqxAB* significantly increased as compared to the parental recipients MICs. In conclusion, plasmid-mediated quinolone resistance linked to *oqxAB* may be a factor in rapid rise in and spread of bacterial quinolone resistance among Egyptian *E. coli* isolates. Chlorpromazine could inhibit efflux pump activity leading to decreased quinolones resistance improving their effectiveness in treatment infectious diseases.

**Supplementary Information:**

The online version contains supplementary material available at 10.1186/s12866-025-03850-7.

## Clinical trial number

Not applicable.

## Introduction

*E. coli* was first identified as the bacterium coli commune by Theodor Escherich in 1885 after being isolated from the feces of newborns and it was named *Escherichia coli* (*E. coli*) later [1]. It has been established that *E. coli* is a normal bacterial species inhabiting the gastrointestinal tracts of warm-blooded animals such as: mammals. Nonetheless, it was discovered that a number of extremely adapted commensal clones were capable of acquiring particular virulence traits, turning them into extremely virulent and frequently fatal pathogens [2].

*E. coli* strains can cause extra-intestinal infections such as: urinary tract infections (UTIs), diverse intra-abdominal, pulmonary, skin and soft tissue infections, newborn meningitis and bacteremia, and intestinal pathologies as well such as: hemolytic-uremic syndrome (HUS). These infections were highly prevalent being associated with high morbidity and mortality. Major HUS epidemics were regularly reported, such as the 2011 epidemic in Europe [3]. Furthermore, antibiotic resistance in *E. coli* increased and it ranked third in the list of (the 12 antibiotic-resistant priority pathogens) described by the WHO in 2017 [4, 5]. The resistance of *E. coli* to various antimicrobials was due to the development of several different mechanisms including: antibiotic inactivation and modifying enzymes, β-lactamases, altered permeability and porin mutations, efflux pumps, and binding site and target mutations.

Fluoroquinolones have antibacterial effect against a broad spectrum of bacteria including Gram positive, Gram negative aerobic bacteria, and some drug-resistant anaerobes [6]. Since 1960, these antibiotics have predominated in the treatment of a variety of infections brought on by *E. coli* [7]. Fluoroquinolone-resistant *E. coli* has grown since 1990 as a result of the increased use of these antibiotics [8].

Resistance for fluoroquinolones has been developed due to the accumulation of point mutations in genes of DNA gyrase in Gram negative bacteria and topoisomerase IV in Gram positive bacteria. This mechanism lowered the affinity of topoisomerase enzymes to bind to quinolones [9] by altering the target site known as the quinolone resistance-determining region (QRDR) [10–12]. The decrease in drug accumulation can also be attributed to the increased drug elimination by multi-drug efflux pumps [7] or down regulation of chromosome-encoded porins. It was also reported that some Gram negative bacteria carried efflux pumps belonging to the resistance nodulation division (RND) superfamily of transporters [13]. Furthermore, plasmid-mediated quinolone resistance (PMQR) genes [14], which are mobile genes resembling plasmids [11], can encode transporters capable of exporting drugs. Efflux pumps carried on plasmids also play a crucial role in bolstering the bacterial resistance against fluoroquinolones [10].

A novel plasmid-encoded multidrug efflux pump OqxAB was detected at the first time in 2004 on the pOLA52 plasmid in *E. coli* strain isolated from swine manure in Denmark [15]. The predominance of *oqxAB* among family Enterobacteriaceae has been increasingly reported over the past years. The overexpression of OqxAB led to resistance for several antibiotics, detergents, and disinfectants and spread of antimicrobial resistance through horizontal transfer among different strains. An in-depth understanding of the epidemiology of oqxaB efflux pump is important for enhancing the antimicrobial use and development of anti-resistance effective medications [15]. The aim of this study was to determine the prevalence of *oqxAB* genes among *E. coli* isolates in Egyptian patients having UTIs and investigate their transferability by conjugation among different strains.

## Materials and methods

### Sample collection, isolation, and identification

One hundred and fifty urine samples were collected from patients with UTIs at Damanhour Medical National Institute, El-Behira, Egypt from April to October 2021. For the detection of *E. coli*, urine samples were cultured on MacConkey agar plates overnight at 37 °C. Gram staining was performed on lactose-fermenting colonies. Several biochemical tests, including triple sugar iron agar, indole, methyl red, Voges Proskauer, and citrate were carried out [16]. *E. coli* isolates were identified at the species level using the automated VITEK 2 system (Bio-Merieux, l’Etoile, France).

### Antibiotic susceptibility testing

The antibiotic resistance of the *E. coli* clinical isolates was determined against thirteen antibiotics representing different antibiotic classes using the standard disc agar diffusion technique according to Bauer et al. [17]. These antibiotics were: ampicillin (AMP 10 µg), chloramphenicol (C 30 µg), amoxicillin/clavulanate (AMC 30 µg), nitrofurantoin (NIT 300 µg), trimethoprim-sulfamethoxazole (COT 25 µg), ciprofloxacin (CIP 5 µg), levofloxacin (LVX 5 µg), gatifloxacin (GAT 5 µg), ceftriaxone (CTR 30 µg), cefazoline (CZ 30 µg), imipenem (IPM 10 µg), amikacin (AK 30 µg), and tetracycline (TE 30 µg) (Oxoid Ltd; Basingostok; Hampshire, England). Diameters of inhibition zones were measured in millimeters (mm), and results were interpreted as susceptible (S), intermediate (I), or resistant (R) based on comparison with the susceptibility tables of the Clinical and Laboratory Standards Institute (CLSI 2021) [18]. Results of the disc diffusion method were confirmed automatically by using the VITEK 2 system (Bio-Merieux, l’Etoile, France).

### Determination of the minimum inhibitory concentration (MIC) of Levofloxacin

MICs of levofloxacin were determined using the broth microdilution method, in accordance with EUCAST/CLSI recommendations. Two fold serial dilutions of levofloxacin ranging from 0.25 to 128 µg/mL were prepared in cation-adjusted Mueller Hinton (MH) broth (HiMedia Laboratories Pvt., Mumbai, India) and inoculated with each *E. coli* isolate. This assay was performed in triplicate for each tested isolate. Bacterial cultures were incubated at 37℃ for 18–20 h and then visually examined for microbial growth to determine the MIC value as the lowest antibiotic concentration that inhibited the growth of the microorganism. The reference breakpoint for the interpretation of MIC against levofloxacin was set as mentioned by CLSI 2021 [18]. MIC ≥ 2 µg/mL was considered resistant. A reference strain *E. coli* ATCC25922 was used as a control.

### Detection of efflux pump-mediated resistance using efflux-pump inhibitor-based microplate assay

Since chlorpromazine (CPZ) was reported to inhibit the efflux-pump in Gram negative bacteria, MIC of CPZ (Sigma Aldrich) was determined for all levofloxacin resistant isolates using the microdilution method in accordance with CLSI 2021 [18]. Half of MIC of CPZ was added to levofloxacin MICs to determine levofloxacin MICs in presence of CPZ, to confirm the presence of efflux activity using broth microdilution. CPZ was used at a concentration of 0.5 MIC to ensure that the efflux pump inhibitor (EPI) would not impair cell viability. It was thought that the presence of the EPI indicated the presence of efflux activity when MIC dropped by at least one-fourth of their initial levels. Each assay was performed in triplicate for each tested isolate [19].

### Molecular detection of *oqxa* and *OqxB* genes using conventional PCR technique

Plasmid DNA of all *E. coli* isolates was extracted using a QIAprep^®^ Spin Miniprep kit (Qiagen, Hilden, Germany) according to manufacture instructions. DNA extracts were tested for the presence of *oqxA* and *oqxB* genes by conventional PCR technique using a thermal cycler (BOECO- BOE8085240, hamburg, Germany) and specific primers (Table 1). Cycling conditions were: initial denaturation at 95 °C for 3 min; followed by 40 cycles of denaturation at 95 °C for 30 s, annealing at 53 °C for 30 s, and extension at 72 °C for 1 min, and a final extension at 72 °C for 10 min. PCR amplicons were then resolved on 1.5% agarose gel stained with ethidium bromide, and visualized via ultraviolet illumination.

**Table 1:** Primers used for detection of *oqxa* and *OqxB* genes using conventional PCR [20]

Gene	Primer sequence (5̕ to 3̕)	Amplicon size (bp)
*oqxA*	*oqxA*-F: CTCGGCGCGATGATGCT *oqxA-*R: CCACTCTTCACGGGAGACGA	392
*oqxB*	*oqxB*-F: TTCTCCCCCGGCGGGAAGTAC *oqxB-*R: CTCGGCCATTTTGGCGCGTA	512

### Transferability of plasmids by conjugation

Ceftriaxone sensitive *E. coli* isolates harboring levofloxacin resistance plasmid were used as plasmid donors. Levofloxacin-sensitive ceftriaxone-resistant *E. coli* isolates were used as recipients. The donor strain was mixed with the recipient one in a ratio of 2: 1. They were spotted onto Luria-Bertani (LB) agar at 28 °C for 16 h after being harvested following centrifugation. 5 mL of LB broth was used to suspend the cells, followed by their culture onto selective and non-selective LB agar plates, and incubation at 37 °C [21]. Selective LB agar plates containing both 2 µg/mL levofloxacin and 4 µg/mL ceftriaxone were used to select the transconjugants. Ceftriaxone-sensitive plasmid-positive isolates and ceftriaxone-resistant recipient cells were selected. Ceftriaxone in the non-selective LB agar plates would kill donor cells, leaving only recipients. Levofloxacin would kill recipient cells except those that have taken the plasmid and developed resistance. Ceftriaxone would kill donor cells on selective plates that contain both antibiotics. Grown colonies were diluted and counted to determine the transfer frequencies by dividing the number of transconjugants by the number of recipient cells [22].

### Sequencing of plasmids of transconjugant and donor isolates

Plasmids were extracted from transconjugant and donor isolates and PCR yields were exposed to electrophoresis in 1.5% agarose gel with 0.5 mg/L of ethidium bromide. After purification using the QIAquick PCR Purification Kit (QIAGEN, Hilden, Germany), they were sequenced using the ABI 3730xl DNA sequencer (Macrogen Inc., Seoul, Korea). Nucleotide sequences were determined for both strands of PCR amplification products. Investigation and comparison of nucleotide sequences was performed using programs accessible at the NCBI (http://www.ncbi.nlm.nih.gov*).*

### Statistical analysis

Statistical analysis was performed by dependent-samples t-test, and effect Size (η2) to detect the correlation between MICs, antimicrobial susceptibility, and PCR results. Spearman correlation coefficient was used. A P value ≤ 0.05 was considered statistically significant.

### Ethical approval

This study adhered to the accepted principles of ethical conduct according to the approval reference number (421PM21) given by the Research Ethics Committee of the Faculty of Pharmacy, Damanhour University. Before testing and molecular analysis of their materials, all the available samples and patient data were gathered with informed ethical consent.

## Results

### Isolation and identification of clinical isolates

One hundred (67%) of the 150 collected clinical isolates were initially identified as *Escherichia* spp. by their growth appearance on MacConkey agar plates and their morphological and biochemical characteristics. Lactose fermenting colonies appeared as Gram negative bacilli. Biochemically, all isolates were indole positive, methyl red positive, Voges Proskauer negative, and citrate negative. On triple sugar iron agar slants, all isolates produced acid butt and slant with gas production without H_2_S production. All samples were confirmed as *E. coli* by using the automated vitek 2 system.

### Antimicrobial susceptibility testing

**Fig. 1:**
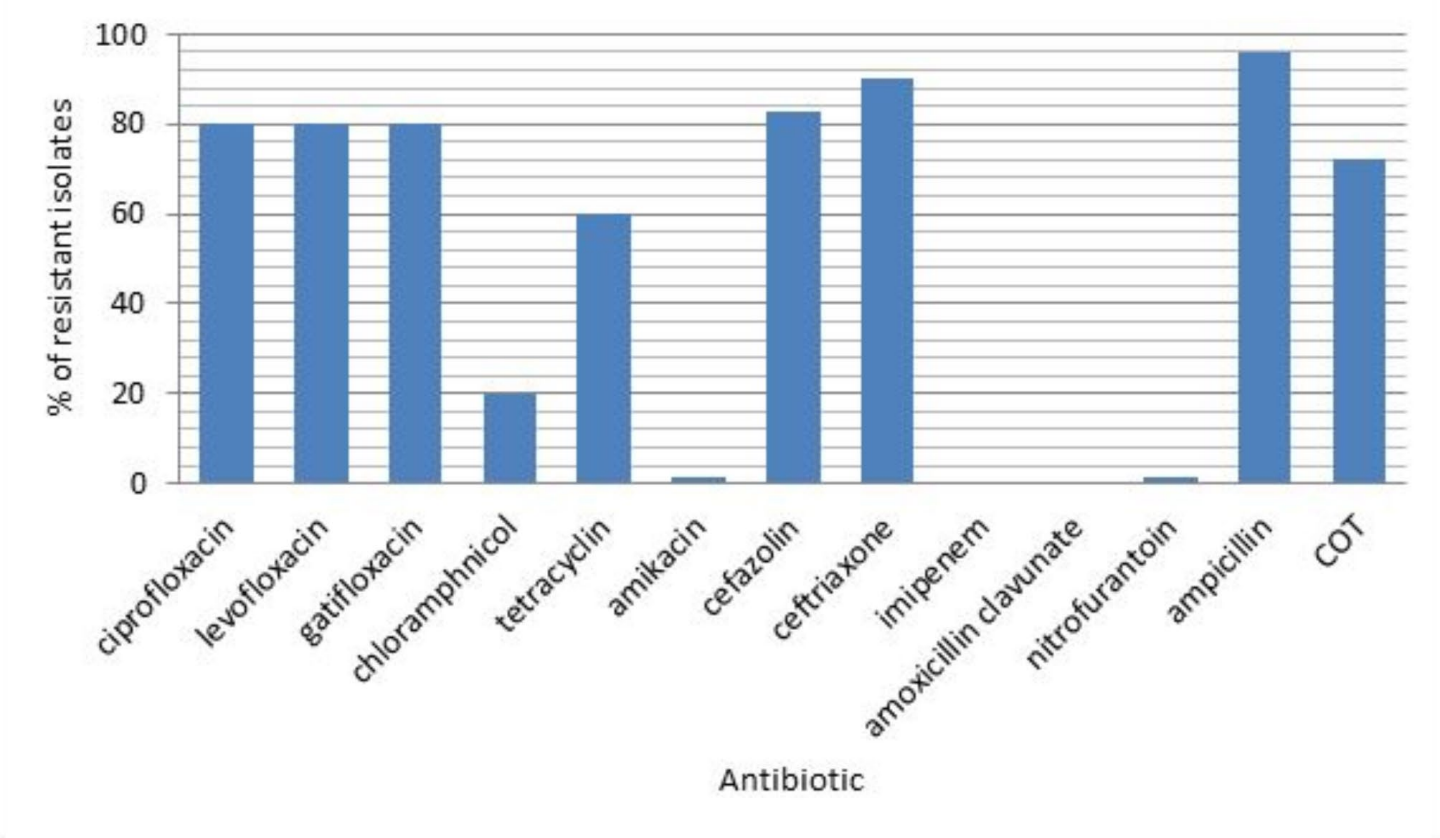
Resistance of *E. coli* isolates against tested antibiotics

Out of the 100 *E. coli* isolates, 80% were resistant to quinolones (ciprofloxacin, levofloxacin and gatifloxacin) and they were multidrug resistant (MDR). The highest resistance percentage was against ampicillin and the lowest resistance percentage was against amikacin and nitrofurantoin (Fig. 1).

### Determination of mics of Levofloxacin and CPZ

**Fig. 2:**
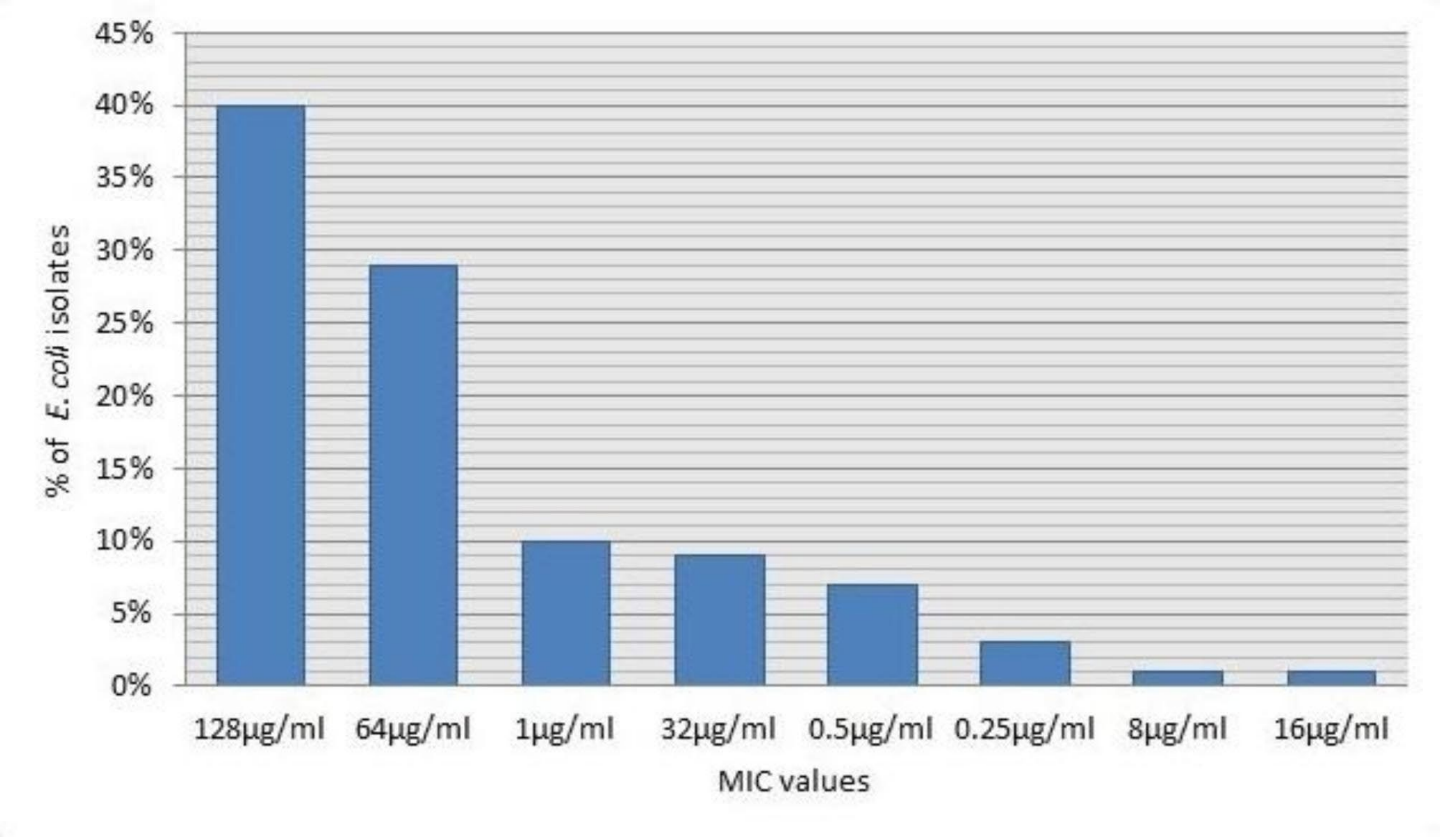
MIC values of levofloxacin among the 80 levofloxacin resistant *E. coli* isolates

MICs of levofloxacin of the 80 levofloxacin resistant *E. coli* isolates were determined using the broth micro-dilution method. MIC values ranged from 0.25 to 128 µg/mL (Fig. 2). Statistically, there was a statistically significant positive correlation (*p* ≤ 0.05) between MICs and antimicrobial susceptibility results of the disc diffusion method.

MIC values of CPZ of the 80 levofloxacin resistant *E. coli* isolates were 64 µg/mL and 32 µg/mL for 87.5% and 12.5% of isolates, respectively. MIC values of levofloxacin in presence of 0.5 MIC of CPZ decreased foR all isolates by 2 to 8 folds (Table 2). Statistically, there was a significant decrease in MICs of levofloxacin in presence of 0.5 MIC of CPZ (*p* = 0.00).

**Table 2:** The effect of efflux pump inhibitor (CPZ) on Levofloxacin resistance among the 80 Levofloxacin resistant *E. coli* isolates

No. of isolates (*n* = 80)	MIC of levofloxacin (µg/mL)	MIC of levofloxacin in presence of 0.5 MIC of CPZ	Fold decrease in MIC of levofloxacin
1	8 (µg/mL)	2 (µg/mL)	**4**
1	16 (µg/mL)	4 (µg/mL)	**4**
9	32 (µg/mL)	8 (µg/mL)	**4**
26	64 (µg/mL)	16 (µg/mL)	**4**
3	64(µg/ml)	8 (µg/mL)	**8**
30	128(µg/ml)	32 (µg/mL)	**4**
7	128 (µg/ml)	16 (µg/mL)	**8**
3	128 (µg/ml)	64 (µg/mL)	**2**

### Molecular detection of *oqxa* and *OqxB* genes using conventional PCR

Forty four (44%) and 39 (39%) isolates were found to harbor *oqxA* and *oqxB* genes, respectively. Statistically, there was a statistically significant positive correlation at the significance level (0.05) between the MIC concentration and the presence of *oqxA* and *oqxB* genes. Besides, there was a statistically significant positive correlation at the significance level (0.05) between disc diffusion test results and presence of *oqxA* and *oqxB* genes.

### Transferability of plasmids by conjugation

To investigate the possibility of horizontal transfer of plasmid-mediated quinolone resistance, conjugation between *E. coli* clinical isolates harboring the *oqxAB* genes as donors and 10 *oqxAB* negative *E. coli* isolates as recipients was performed. Ceftriaxone was the antimicrobial agent used for selection. A striking increase of levofloxacin MIC was observed for all recipient isolates after conjugation (Table 3).

**Table 3:** Plasmid transfer frequencies and mics of Levofloxacin of transconjugants and recipient isolates

Recipient isolates (*n* = 10)	*Conjugation frequency	MIC (µg/mL) of LEV of recipient isolates	MIC (µg/mL) of LEV of transconjugants
2 samples	5 × 10^− 7^	1	128
1 samples	1 × 10^− 4^	0.5	32
2 samples	**4 × 10** ^**− 3**^	**0.5**	**8**
5 samples	**1.6 × 10** ^**− 4**^	**0.25**	**16**

*Conjugation frequency = number of transconjugants/number of donor cells

### Sequencing

**Fig. 3:**
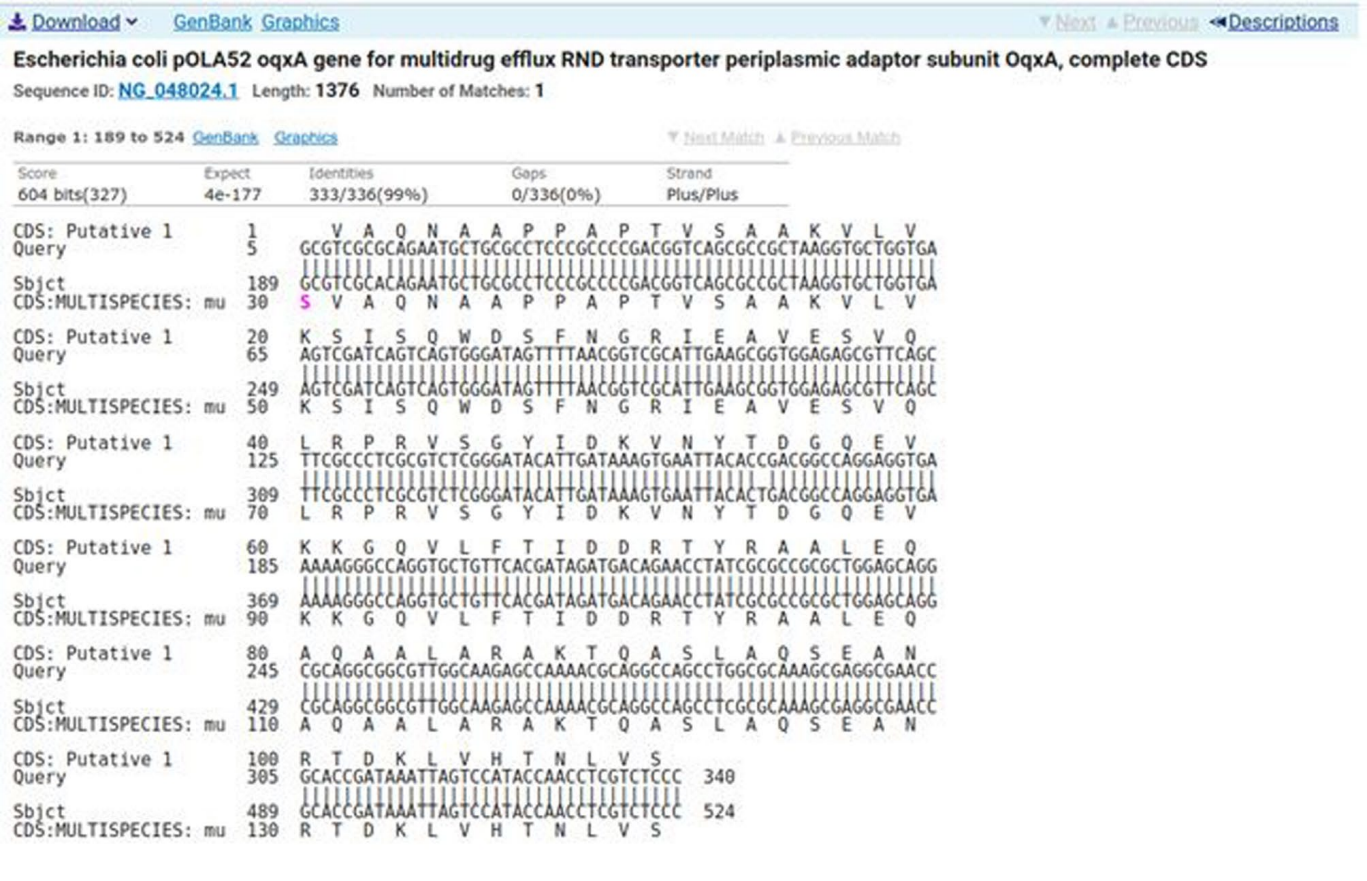
BLAST alignment of the sequence of Sample_29A_AF against the reference NG_048024.1

**Fig. 4:**
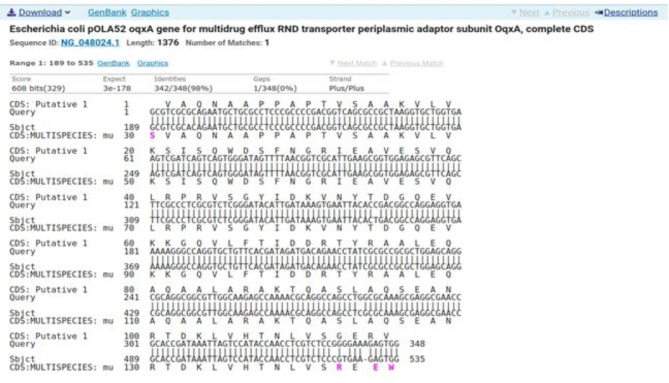
BLAST alignment of the sequence of Sample_36A_AF agianst the reference NG_048024.1

**Fig. 5:**
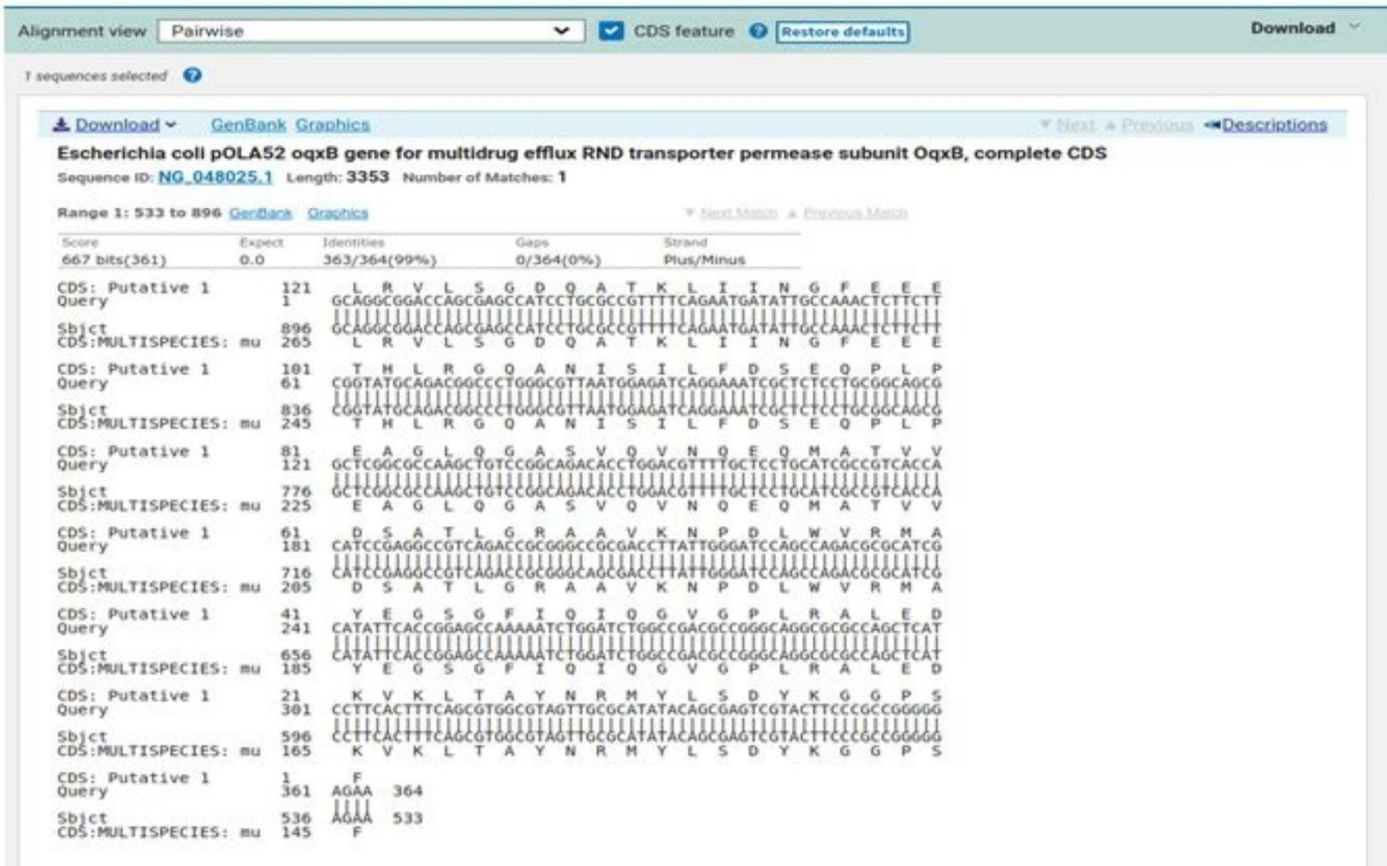
BLAST alignment of the sequence of sample_29B_BR against the reference NG_048025.1

**Fig. 6:**
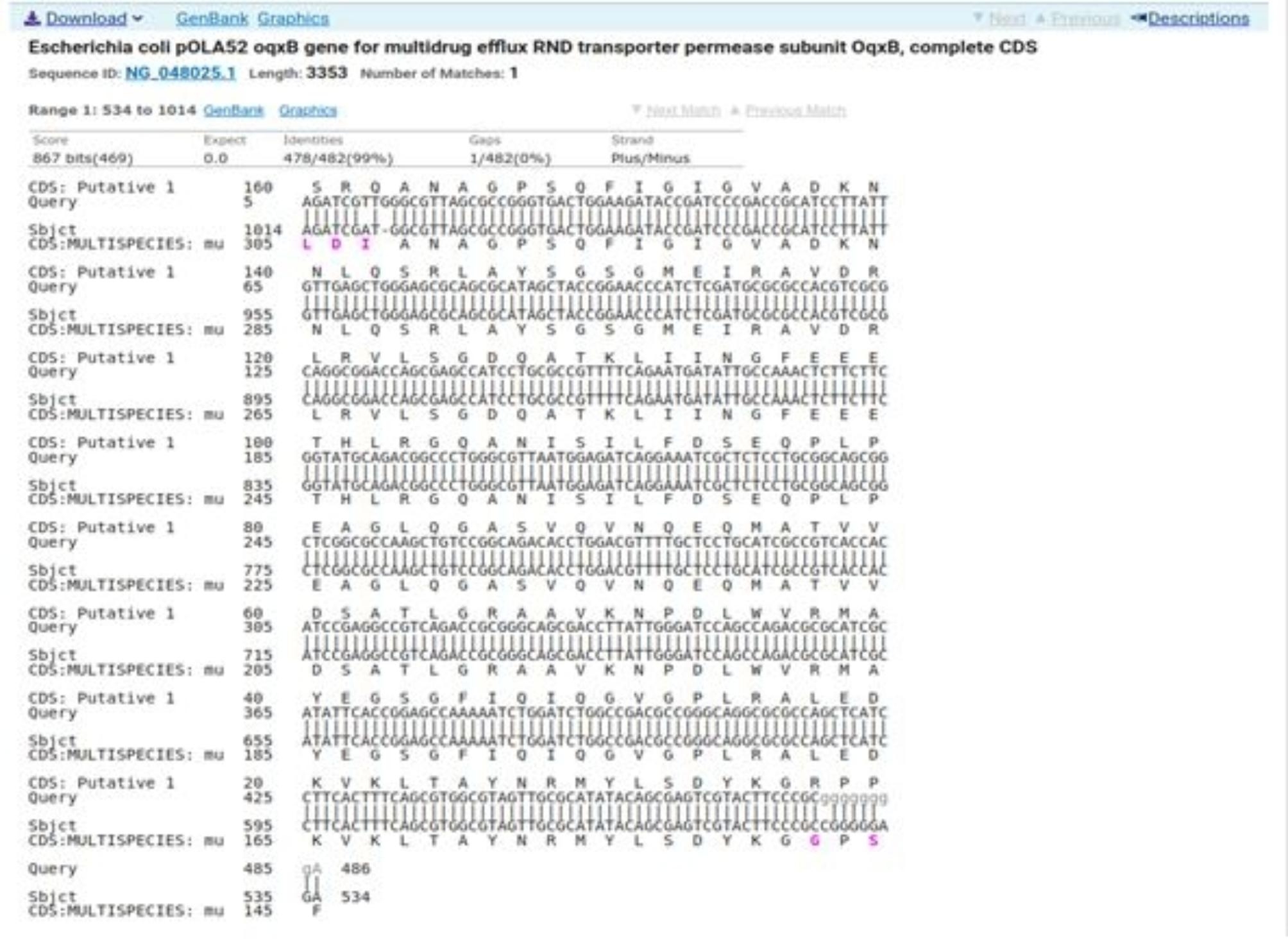
BLAST alignment of the sequence of sample_36B_BR against the reference NG_048025.1

**Fig. 7:**
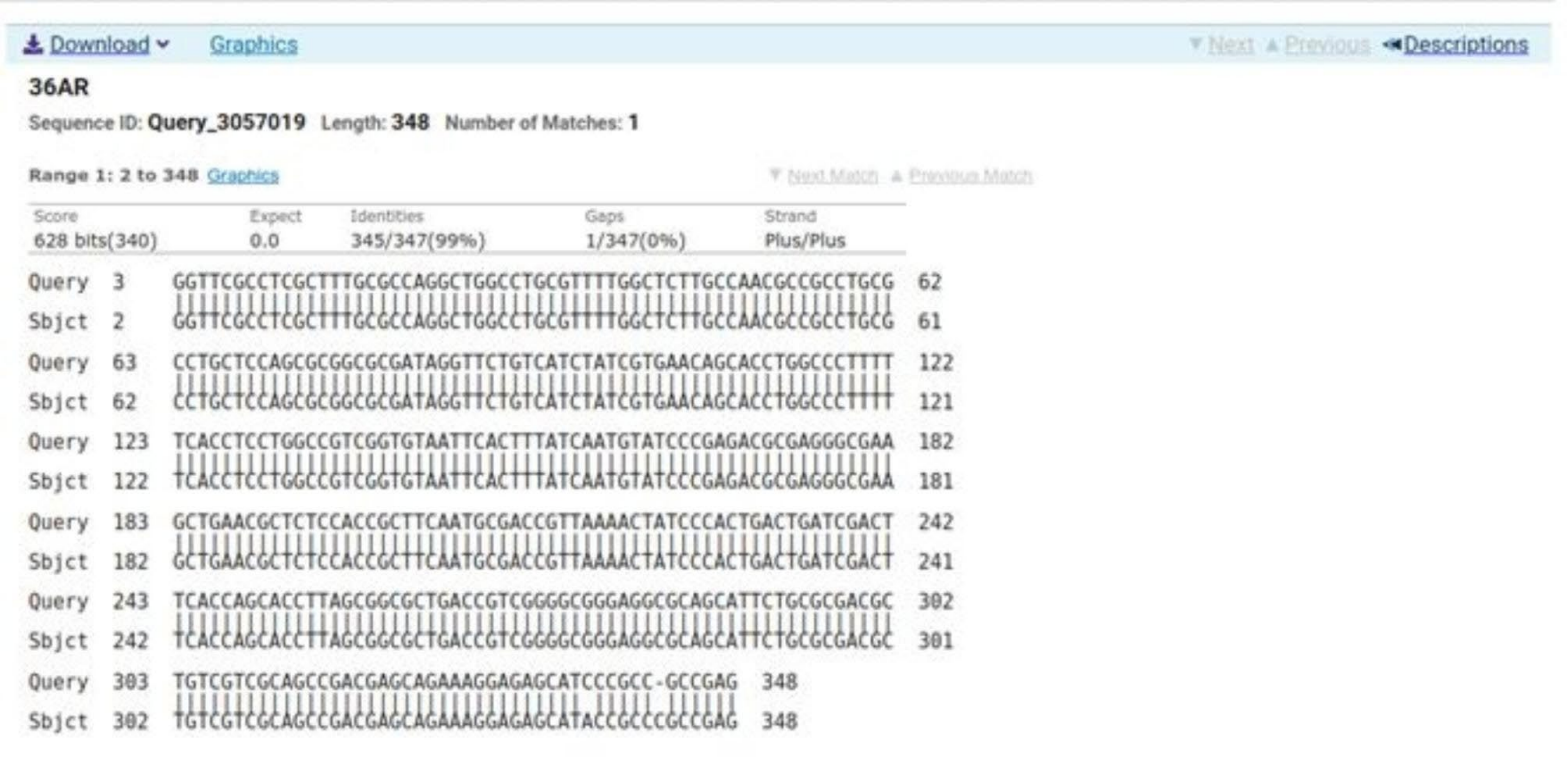
BLAST alignment of the sequence of Sample_29A_AR against sample_36A_AR

**Fig. 8:**
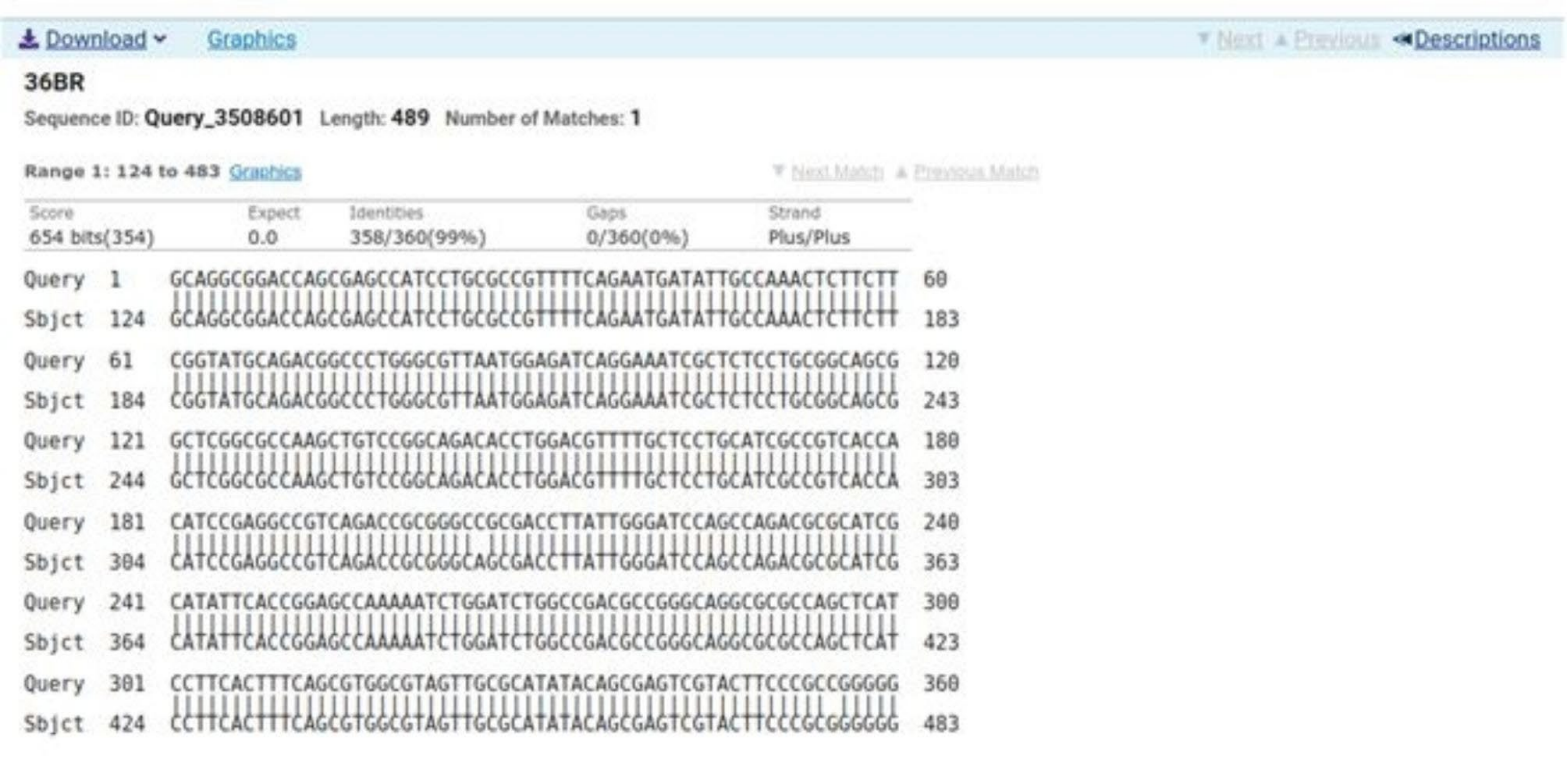
BLAST alignment of the sequence of sample_29B_BR against Sampel_36B_BR. Nucleotide sequence accession number

Sequencing was performed for two tested isolates (Isolate no. 29 as donor and isolate no. 36 as recipient) using the ABI 3730xl DNA sequencer. Sequence alignment of E29 *oqxA* and E36 *oqxA* with the reference NG_048024.1 showed 99% and 98% identity, respectively with changes in 3 amino acids in the latter (Figs. 3 and 4). Sequence alignment of E29 *oqxB* and E36 *oqxB* against with the reference NG_048025.1 showed 99% identity for both isolates with changes in 5 amino acids in the latter (Figs. 5 and 6). Alignment of E29 with E36 for *oqxA* gene had 99% identity (Fig. 7) and alignment of E29 with E36 for *oqxB* gene showed 99% identity (Fig. 8). The datasets generated and/or analyzed during the current study are available in the GenBank repository. Accession numbers PP502431 and PP502432 were deposited for *oqxA* and *oqxB*, respectively.

## Discussion

Fluoroquinolones are antibiotics used frequently for treatment of UTIs. More than 50% of *E. coli* infections in numerous countries worldwide no longer respond to fluoroquinolone treatment [23]. In this study, it was found that 96% of tested *E. coli* isolates were resistant to β-lactam antibiotics. A lower resistance percentage (90%) was reported by Hassuna et al. [24]. In this study, only 5% of our isolates were resistant to amoxicillin/clavulanate; however, a higher resistance percentage (39.2%) was reported by Vanstokstraeten et al.. in Belgium [25]. In this study, all *E. coli* isolates were susceptible to imipenem and resistant to ampicillin; these findings were in line with those reported by *Mendonca et al.* in Portugal [26]. Our results were also consistent with *Ait-Mimoune et al.*. as an absence of resistance for imipenem was reported in Algeria [27]. In addition, *Demir et al.* reported low resistance rates of *E. coli* for imipenem with a value of 1.4% in Turkey [28].

Regarding fluroquinolones, 80% of our isolates were resistant to ciprofloxacin, levofloxacin and gatifloxacin by the disc diffusion method in this study. In contrast, Zaki et al.. reported that 52.3% of their isolates were resistant to ciprofloxacin [29]. In addition, *Deku et al.*. reported that 51.1% and 35.7% of their isolates were resistant to ciprofloxacin and levofloxacin, respectively [30]. On the other hand, Esmaeel et al. reported fluroquinolones resistance among all (100%) their *E. coli* isolates [31]. Moreover, Mohamed et al.. reported that 25% of their isolates exhibited susceptibility to levofloxacin [32]; these results were in accordance with ours as 20% of our isolates were susceptible to levofloxacin. The variability in susceptibility of *E. coli* clinical isolates toward levofloxacin in different Egyptian studies may be due to differences in geographical zones or using different protocols of antibiotics.

In this study, all quinolone resistant *E. coli* isolates were MDR (resistant to one or more drugs in three or more antibacterial classes) [33]. There are numerous explanations for the increased prevalence of MDR in *E. coli* isolates. Plasmids carrying MDR genes, including quinolones, are a main obvious cause. Similar to our results, Majlesi et al. reported that fluroquinolone resistant *Enterobacteriaceae* isolates showed MDR to other antimicrobial agents in Iran [34].

In this study, MIC values of levofloxacin ranged from 0.25 to 128 µg/mL; on the other hand, Hassan et al.. reported that their MIC values ranged from 8 to 32 µg/mL [35]. OqxAB was encoded by *oqxA* and *oqxB* genes located on a52-kb conjugative plasmid designated pOLA52 and conferred resistance to multiple agents, including fluoroquinolones [36, 37].One of the most important weapons for promoting bacterial survival is the efflux pump which extrudes harmful substances out of cells decreasing antibiotic intracellular concentration [38]. This explains that MIC values of our isolates carrying *oqxAB* genes were higher than those negative for these efflux genes. In this study, we investigated the association of the oqxaB efflux pump with resistance to fluoroquinolones. PCR results showed that 44% of our isolates carried *oqxA* and 39% carried *oqxB* genes. Most of *oqxAB* positive isolates were resistant to ciprofloxacin, levofloxacin and gatifloxacin with MICs ≥ 64 µg/mL. *oqxAB* genes were first reported in Egypt by Haggag et al. [19] as 5.9% of their isolates contained them. Another Egyptian study reported that 72.22% of their isolates contained *oqxAB* and *qepA* genes [39]. In contrast, a low prevalence (18.07%) of *oqxAB* genes was reported by Wang et al. [40]. Similar to our results, Gabr et al. [41]. and Liu et al. [42]. reported that both *oqxA* and *oqxB* genes were detected in 43.7% and 42% of their MDR *E. coli* isolates, respectively.

One promising strategy to combat bacterial multidrug resistance is the administration of efflux pump inhibitors, which reduce the functionality of these pumps [43, 44]. They have garnered significant interest due to their strong ability to modify resistance and restore the diminishing therapeutic effectiveness of existing antibiotics. By preventing calcium from entering calcium-dependent ATPase, CPZ reduces the amount of produced protons, which is necessary for the primary myelofibrosis to be maintained. In this study, there were significant differences in MIC values of levofloxacin in presence of CPZ and the effect size (η2) of CPZ in decreasing MIC values was 0.827. The percentage of change in MIC of levofloxacin due to the addition of CPZ was 82.7%. In this study, CPZ reduced MICs of levofloxacin to one-fourth of their original values and our results were in accordance with Helmy et al. and Martins et al. studies [19, 45]. On the other hand, Chowdhury et al. reported that the presence of omeprazole as efflux pump inhibitor increased susceptibility of every MDR *E. coli* isolate to at least 1 of the 7 investigated antibiotics [46].

In order to test the plasmid’s conjugal transfer ability, ten *E. coli* isolates were used as recipients in this study. The plasmid’s conjugation transfer frequencies varied among recipients. Levofloxacin MICs for all transconjugants were comparable to those of donors, and were noticeably higher than recipients. In this study, *oqxA* and *oqxB* genes were confirmed by conjugation experiments to be located on transferable plasmids. In contrast to our results, Kim et al. reported that no direct transfer or co-transfer of *oqxAB* genes was detected [47]. In agreement with our results, Zhao et al., Basu et al.., Ho et al. and Wong et al. reported that *oqxAB* genes were located on transferable plasmids [48–51]. In addition, 8 transconjugants were reported to acquire *oqxAB* genes through conjugation by Liu et al. [42].

In this study, sequencing of *oqxAB* positive transconjugants isolates was performed. High similarity was found between them and the original *oqxA* and *oqxB* sequences of pOLA52. The presence of these genes in transconjugants provided evidence that the transmission and reassembly of *PMQR* genes resulted in formation of resistance plasmids [52]. The transmission of conjugative plasmids encoding levofloxacin resistance among different isolates was expected to impair the effectiveness of fluoroquinolone antibiotics in treating bacterial infections. This outcome was consistent with other studies [51, 52]. According to sequencing results, changes in gene sequence of isolate36 (transgonjugant) could be due to the gene transfer process showing missense mutation that didn’t affect the functionality of genes when aligned with NCBI reference sequences.

In conclusion, high prevalence of *oqxAB* plasmid mediated quinolone resistance was detected in *E. coli* isolates recovered from UTIs in Egypt. The efflux pump inhibitor, CPZ, inhibited efflux pump activity in all isolates of the current study leading to decreasing quinolones resistance improving their effectiveness in treatment of infectious diseases.

## Electronic supplementary material

Below is the link to the electronic supplementary material.

Supplementary Material 1

## Data Availability

The datasets generated and/or analyzed during the current study are available in the GenBank repository. Accession numbers PP502431 and PP502432 were deposited for oqxA and oqxB, respectively.
