# Supplementary material for: Chlorpromazine inhibits the plasmid-mediated oqxAB multidrug efflux pump in Escherichia coli isolates of Egyptian patients with utis
Source: BMC Microbiol. 2025 Mar 26;25:171. doi: 10.1186/s12866-025-03850-7 (PMC11938646; doi:10.1186/s12866-025-03850-7)
Supplement: Supplementary file 1 — Supplementary Material 1 [file 12866_2025_3850_MOESM1_ESM.docx]

To detect the relationship between the MIC concentration and each of the (S/R) and genes, the researcher used the Spearman correlation coefficient, and the results are shown in the following table:

**Table (***) Correlation coefficients between MIC concentration and each of the (S/R) and genes (n=100)**

|  | **MIC concentration** | | **S/R** | | | **genes** | | |
| --- | --- | --- | --- | --- | --- | --- | --- | --- |
| **MIC concentration** | **-----** | | **.726^**^** | **P=0.005** | | **.692^**^** | **P=0.001** | |
| **S/R** | **.726^**^** | **P=0.0001** | **------** | | | **.493^**^** | | **P=0.0001** |
| **genes** | **.692^**^** | **P=0.001** | **.493^**^** | | **P=0.003** | **-----** | | |
| **** Spearman Correlation coeffecient** | | | | | | | | |

It is clear from the previous table that:

- There is a statistically significant positive relationship at the significance level (0.05) between the MIC concentration and (S/R).
- There is a statistically significant positive relationship at the significance level (0.05) between the MIC concentration and the genes.
- There is a statistically significant positive relationship at the significance level (0.05) between (S/R) and genes.
